# Retrospective analysis of insulin responses to standard dosed oral glucose tests (OGTs) via naso-gastric tubing towards definition of an objective cut-off value

**DOI:** 10.1186/s13028-018-0358-8

**Published:** 2018-01-19

**Authors:** Tobias Warnken, Julien Delarocque, Svenja Schumacher, Korinna Huber, Karsten Feige

**Affiliations:** 1 Clinic for Horses, University of Veterinary Medicine Hannover, Foundation, Bünteweg 9, 30559 Hannover, Germany; 2 Institute of Animal Science, Faculty of Agricultural Sciences, University of Hohenheim, Fruwirthstraße 35, 70593 Stuttgart, Germany

**Keywords:** ELISA, Equine metabolic syndrome, Horse, Insulin, Insulin dysregulation, Naso-gastric tubing, Oral glucose test

## Abstract

**Background:**

Insulin dysregulation (ID) with basal or postprandial hyperinsulinemia is one of the key findings in horses and ponies suffering from the equine metabolic syndrome (EMS). Assessment of ID can easily be performed in clinical settings by the use of oral glucose challenge tests. Oral glucose test (OGT) performed with 1 g/kg bodyweight (BW) glucose administered via naso-gastric tube allows the exact administration of a defined glucose dosage in a short time. However, reliable cut-off values have not been available so far. Therefore, the aim of the study was to describe variations in insulin response to OGT via naso-gastric tubing and to provide a clinical useful cut-off value for ID when using the insulin quantification performed with an equine-optimized insulin enzyme-linked immunosorbent assay.

**Results:**

Data visualization revealed no clear separation in the serum insulin concentration of insulin sensitive and insulin dysregulated horses during OGT. Therefore, a model based clustering method was used to circumvent the use of an arbitrary limit for categorization. This method considered all data-points for the classification, taking into account the individual insulin trajectory during the OGT. With this method two clusters were differentiated, one with low and one with high insulin responses during OGT. The cluster of individuals with low insulin response was consistently detected, independently of the initialization parameters of the algorithm. In this cluster the 97.5% quantile of insulin is 110 µLU/mL at 120 min. We suggest using this insulin concentration of 110 µLU/mL as a cut-off value for samples obtained at 120 min in OGT.

**Conclusion:**

OGT performed with 1 g/kg BW glucose and administration via naso-gastric tubing can easily be performed under clinical settings. Application of the cut-off value of 110 µLU/mL at 120 min allows assessment of ID in horses.

## Findings

The equine metabolic syndrome (EMS) is a common endocrinopathy in equines. Horses are affected by general or regional obesity, predisposition to laminitis and insulin dysregulation (ID). Insulin dysregulation refers to basal and/or postprandial hyperinsulinemia, sometimes also associated with tissue insulin resistance [1, 2]. Moreover, ID can occur in EMS as well as in pituitary *pars intermedia* dysfunction (PPID) patients [3, 4]. Unfortunately, ID horses may not be identified correctly by phenotype in all cases. Furthermore, baseline measurements of fasting glucose and insulin concentrations may not suffice in all patients [1, 5]. Therefore, dynamic tests are proposed for the assessment of ID [1, 2, 6, 7].

Oral glucose challenge tests allow assessment of postprandial hyperinsulinemia in ID horses under standardized conditions. Recently published research highlights the importance of hyperinsulinemia, reporting that laminitis can be induced experimentally using a diet high in nonstructural carbohydrates. Furthermore, the authors were able to predict laminitis risk based on insulin and glucose levels after an oral challenge test [8]. Several oral test protocols for assessment of ID are currently available. In these procedures application routes as well as the used dosages of glucose or other sugars vary. The in-feed oral glucose tests (OGT) can be performed by feeding 0.5 g–1.0 g/kg bodyweight (BW) glucose or dextrose powder mixed in low-glycemic meals and by determination of insulin and glucose concentrations after 120 min [9]. A positive test and ID was defined as an insulin concentration > 80 µLU/mL [1] or > 87 µLU/mL [10], depending on which literature is consulted.

The oral sugar test (OST) as a simplified testing procedure uses 0.15 mL/kg BW corn syrup [11] administered orally via syringe, followed by measurement of insulin and glucose after 75 min. Insulin concentrations > 60 μLU/mL were used as cut-off [10, 12]. Recently, a dosage of 0.25 mL/kg BW for OST was suggested to improve diagnostic value. Blood sample analysis was recommended at either 60, 75, 90 or 120 min and insulin concentrations of ≥ 22.8, 18.7, 30.2 and 26.3 µLU/mL, respectively were proposed for being indicative for ID [13]. Since corn syrup is not available in most European countries, a modified OST using commercially available Scandinavian glucose syrup was developed and provided promising results [14]. Nevertheless, reference ranges have not been established to date. Moreover, clinicians and researchers reported acceptance problems in their patients when performing in-feed OGTs, which led to prolonged consumption times or refused feed intake and therefore precluded reliable and exact test results for interpretation [15].

An alternative is to perform the OGT via naso-gastric tubing [16]. The substantial benefit of this protocol is the exact administration of a defined glucose dosage in a short time. Though it remains the most precise oral test approach, this procedure requires naso-gastric tubing.

For OGT performed with 1 g/kg BW glucose dissolved in 2 L water and administration via naso-gastric tubing, there are no reliable cut-off values or reference ranges available. Therefore, the aim of the study was to describe variations in insulin response to OGT via naso-gastric tubing and to provide a clinical useful cut-off value for ID when using insulin quantification performed with an equine-optimized insulin enzyme-linked immunosorbent assay (ELISA).^1^

OGT results of 56 horses and ponies were obtained under similar conditions during several research projects from winter 2013 to spring 2017. Twenty-three warmblood horses, 19 Icelandic horses, 5 Shetland ponies and 9 ponies of various breeds were included in the study regardless of their insulin sensitivity status. There were 26 mares, 25 geldings and 5 stallions, aged 15 ± 6 years and weighed 473 ± 136 kg. The included horses had a mean body condition score (BCS) of 5.9 ± 1.4. Out of 56 individuals 16 were previously diagnosed with laminitis. OGTs were performed under standardized conditions after 12–14 h fasting prior to testing.

To perform OGT, 1 g/kg BW glucose powder^2^ was dissolved in 2 L of water and administered via naso-gastric tubing [16]. Blood samples were collected via intravenous catheter^3^^,^^4^ prior to administration of the glucose solution, and afterwards in 15 min intervals for at least 180 min. Blood samples for serum preparation were placed into plain tubes, incubated at room temperature, centrifuged after 60 min at 1000×*g* for 6 min, and stored at − 80 °C until further analysis. Serum insulin concentrations were analyzed in duplicate using an equine-optimized insulin ELISA (see footnote 1) previously validated for use in horses [17, 18]. Samples with insulin concentrations exceeding the analytical range of the ELISA (> 1.5 µg/L) were diluted with commercially available sample buffer.^5^ For the conversion of insulin concentrations expressed in µg/L as supplied by the ELISA, to the commonly used SI unit of µLU/mL, the previously published conversion factor of 115 was used [19].

**Fig. 1:**
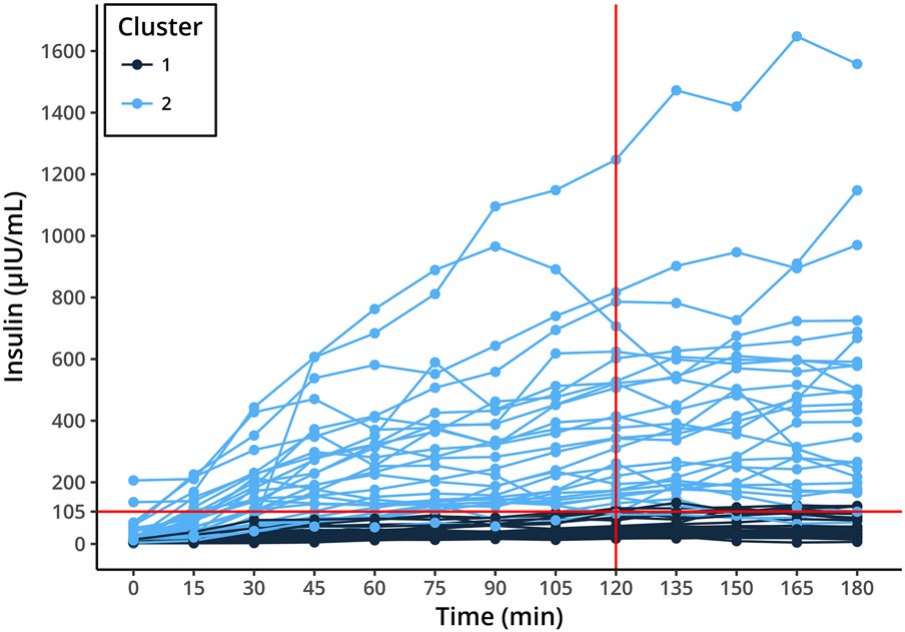
Serum insulin concentrations during oral glucose test (OGT), n = 56. Model based clustering algorithm detected an intrinsic structure to the data and grouped individuals based on their similarities in all insulin measurements in an unsupervised manner. This figure shows the two clusters detected by the algorithm (cluster 1—dark blue; cluster 2—light blue). The calculated limit between both clusters at 120 min was 105 µLU/mL (red lines)

**Fig. 2:**
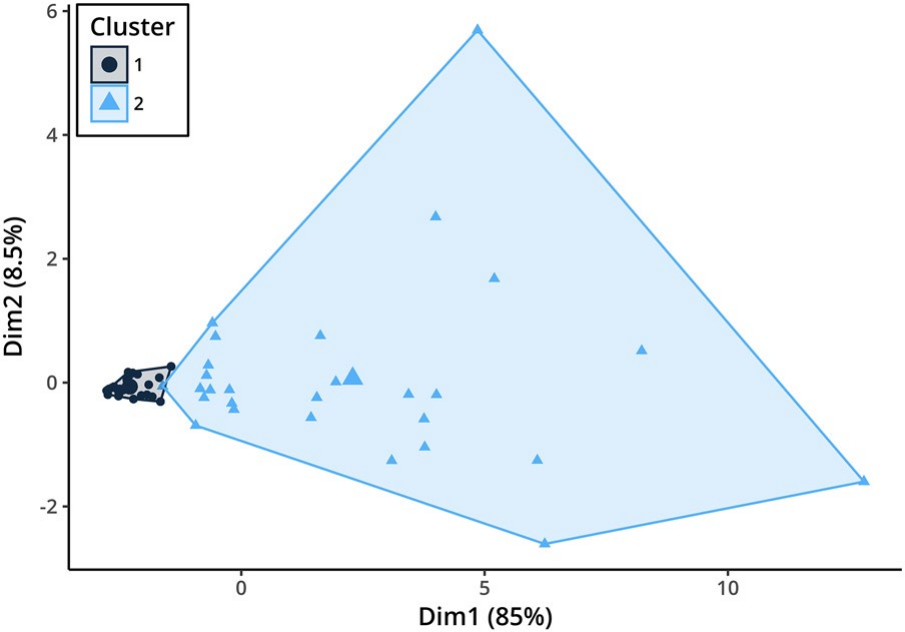
Principal component analysis (PCA) plot of the two clusters. A PCA plot is a 2D representation of high-dimensional data. In this case, the dimensions consist in measurements of serum insulin at different time points of the oral glucose test (OGT). Data-points that are close show a similar insulin response during the OGT (cluster 1—dark blue points; cluster 2—light blue triangles)

**Fig. 3:**
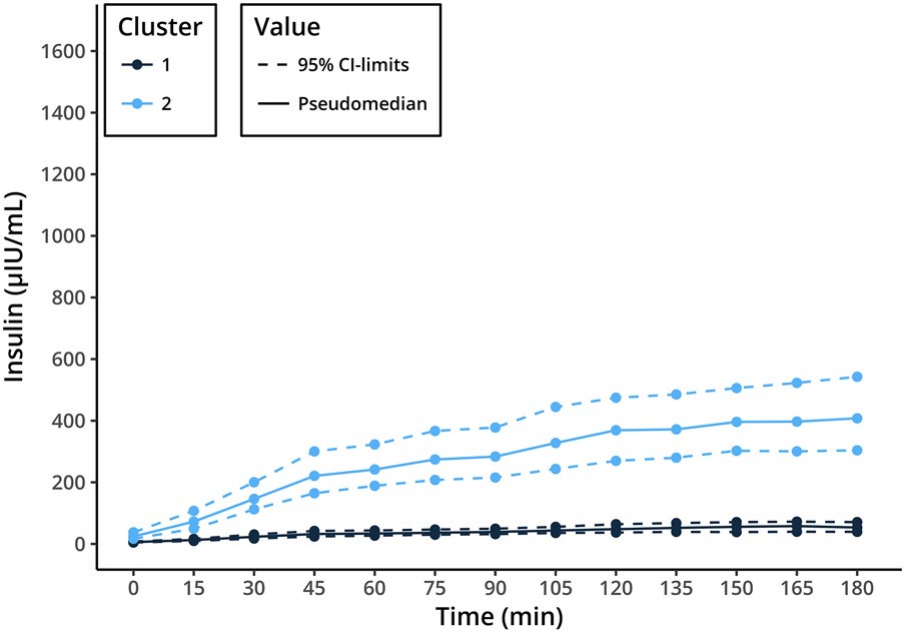
Pseudomedian (solid line) and 95% confidence interval (dashed line) of the insulin response in oral glucose test (OGT) for both clusters (cluster 1—dark blue; cluster 2—light blue). As the distribution of the insulin levels in each cluster for each time-point are not normal, the median was chosen as a better representation of how the insulin response differs between the clusters

**Fig. 4:**
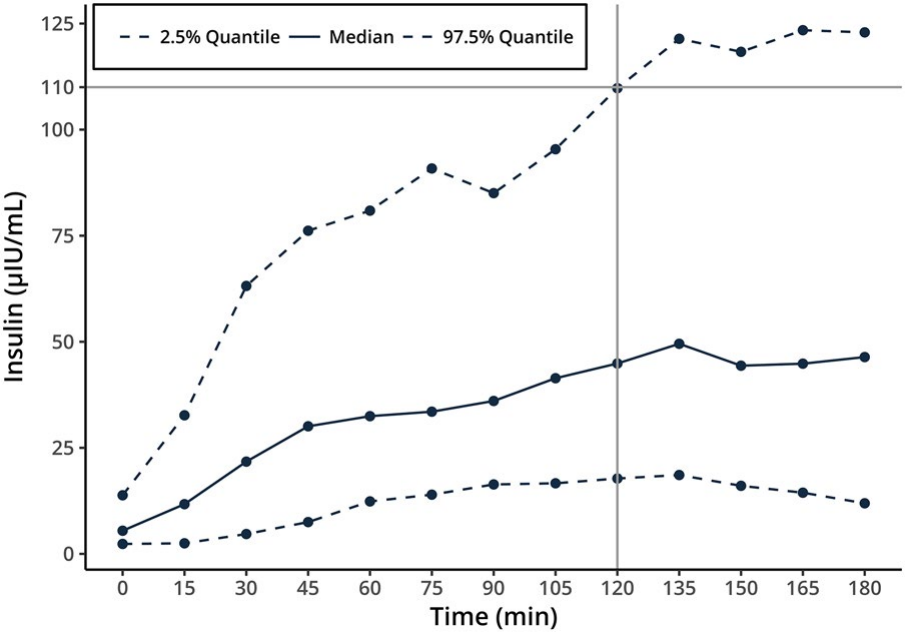
Median serum insulin concentration (solid line) of the cluster 1 classified as insulin sensitive during oral glucose test (OGT) with calculated 2.5 and 97.5% quantile (dashed lines). Calculated cut-off at 120 min is 110 µLU/mL (grey lines)

Statistical analysis was performed in R 3.4.0.^6^ Dynamics in insulin responses to OGT showed large variation in the study population ruling out simple visual differentiation between two groups of insulin sensitive and insulin dysregulated animals (Fig. 1). Moreover, there is no clear separation in the serum insulin concentration of insulin sensitive and insulin dysregulated horses at the currently used time-point for evaluation of 120 min in the OGT. Therefore, data analysis was performed using a model based clustering method provided by the mclust R-package [20] in combination with a scaled singular value decomposition (SVD) projection for improved initialization [21]. This algorithm tries to detect an intrinsic structure to the data in an unsupervised manner by grouping individuals based on their similarities in insulin measurements at all sampling time points. The benefits are that no arbitrary limit is used for categorization and that all data-points are used in the classification, taking into account the individual insulin response during the OGT. The clustering results were compared with other clustering methods like partitioning (kmeans, pam) and hierarchical clustering with varying initialization parameters. A cluster with constant lower insulin concentrations during the OGT (cluster 1) was consistently found across the different clustering strategies, while the optimal number of clusters varied. The model based clustering approach was preferred because it required no a priori estimation of the number of clusters. Two clusters retained by the mclust algorithm with improved initialization relate to another (Figs. 1, 2). The separation line between the two clusters at 120 min was at 105 µLU/L insulin. As the serum insulin in each group for each time-point did not belong to a normal distribution, the (pseudo) median was of more informative value than the mean. Figure 3 shows the pseudomedian with 95% confidence interval for both clusters as estimated from the Hodges-Lehmann estimator. While the difference between groups was striking, the actual range of insulin responses that occurred in different subjects supports the idea that insulin status is not a dichotomous state of either insulin sensitive or insulin dysregulated but rather that ID exists in different intensities (Fig. 1). For calculation of a reliable cut-off value, 97.5% quantile of the cluster 1 constituted a cut-off of 110 µLU/mL insulin at 120 min (Fig. 4). With respect to previously reported significant differences between laboratory methods used for quantification of equine insulin [18, 22, 23], our reported cut-off value is only applicable for the combination of the OGT procedure described above and the measurement of insulin by the use of the equine-optimized ELISA (see footnote 1) or a method which shows good agreement. However, even intra- and inter-assay CV values of 2.0–10.6% and 4.83–10.7% reported for the immunoassay used in this study can impair final results [17, 18, 24]. Sixteen horses of the study population had radiographically confirmed laminitis. Fifteen out of sixteen laminitic horses and ponies were positively identified as ID by this OGT when the cut-off value of 110 µLU/mL was applied. Moreover, the median (IQR) insulin concentration of previously laminitic individuals was 513.3 (187.6–618.9) µLU/mL at 120 min during OGT. This is the first study providing a cut-off for OGT performed with 1 g/kg BW glucose administered via naso-gastric tube. Nevertheless, insulin concentrations around 110 µLU/mL should be interpreted carefully in clinical situations in which significant management and feeding modifications or even drug therapies would be initiated when patients are classified as insulin dysregulated. In these cases with debatable insulin concentrations following OGT, re-testing horses and ponies after a certain period of time would be advisable. The authors suggest not using the reported value as a strict cut-off, but rather as an orientation value for clinical interpretation and diagnosing ID because of the fluent transition between horses and ponies with undisturbed insulin regulation and ID.

Taking into account the reported coefficient of variation (CV) values of 19% (31–43%) for in-feed OGTs at 120 min [15] and 83–91% agreement between OST performed on different days [25], multiple factors can affect final test results. Furthermore, interpretation of OGT insulin concentrations should be accompanied by consideration of clinical findings when assessment of ID is used for diagnosing EMS or PPID related ID. Fluent transition from an insulin sensitive state to an insulin dysregulated state complicates the establishment of reliable cut-offs. However, it emphasizes the importance of early detection of horses being at high risk to develop metabolic pathologies and the importance of recording the degree of severity when individual therapy plans are created.

In conclusion, the standard dosed OGT with glucose application via naso-gastric tubing can be easily performed under clinical settings and allows reliable assessment of ID in horses.

## Abbreviations

BW: bodyweight
CV: coefficient of variation
ELISA: enzyme-linked immunosorbent assay
EMS: equine metabolic syndrome
Fig: figure
ID: insulin dysregulation
IQR: interquartile range
IS: insulin sensitivity
OGT: oral glucose test
OST: oral sugar test
PPID: pituitary *pars intermedia* dysfunction
SVD: singular value decomposition

## Footnotes

1 Equine Insulin ELISA, Mercodia AB, Uppsala, Sweden.

2 Glukose, WDT, Garbsen, Germany.

3 EquiCath^TM^ Fastflow, 12 G, Braun Vet Care GmbH, Tuttlingen, Germany.

4 Intraflon 2, 12 G, Vygon, Ecouen, France.

5 Diabetes Sample Buffer, Mercodia AB, Uppsala, Sweden.

6 R version 3.4.0, The R Foundation for Statistical Computing.

## References

[1] Bertin FR, de Laat MA (2017). The diagnosis of equine insulin dysregulation. Equine Vet J.

[2] The Equine Endocrinology Group (EEG). Recommendations for the diagnosis and treatment of equine metabolic syndrome (EMS). https://sites.tufts.edu/equineendogroup/files/2016/11/2016-11-2-EMS-EEG-Final.pdf. Accessed 15 Dec 2016.

[3] McFarlane D (2011). Equine pituitary pars intermedia dysfunction. Vet Clin North Am Equine Pract..

[4] The Equine Endocrinology Group (EEG). Recommendations for the diagnosis and treatment of pituitary pars intermedia dysfunction (PPID). 2015. https://sites.tufts.edu/equineendogroup/files/2015/12/2015-10-16_EEG-2015-recommendations.pdf. Accessed 15 Dec 2016.

[5] Pratt SE, Siciliano PD, Walston L (2009). Variation of insulin sensitivity estimates in horses. J Equine Vet Sci..

[6] Firshman AM, Valberg SJ (2007). Factors affecting clinical assessment of insulin sensitivity in horses. Equine Vet J.

[7] Frank N, Tadros EM (2014). Insulin dysregulation. Equine Vet J.

[8] Meier AD, de Laat MA, Reiche DB, Pollitt CC, Walsh DM, McGree JM, Sillence MN (2018). The oral glucose test predicts laminitis risk in ponies fed a diet high in nonstructural carbohydrates. Domest Anim Endocrinol.

[9] Smith S, Harris PA, Menzies-Gow NJ (2016). Comparison of the in-feed glucose test and the oral sugar test. Equine Vet J.

[10] Frank N, Geor R (2014). Current best practice in clinical management of equine endocrine patients. Equine Vet Educ..

[11] Schuver A, Frank N, Chameroy KA, Elliott SB (2014). Assessment of insulin and glucose dynamics by using an oral sugar test in horses. J Equine Vet Sci..

[12] Frank N (2011). Equine metabolic syndrome. Vet Clin North Am Equine Pract..

[13] Manfredi JM. Identifying breed differences in insulin dynamics, skeletal muscle and adipose tissue histology and biology. Dissertation, Michigan State University, 2016.

[14] Lindase S, Nostell K, Brojer J (2016). A modified oral sugar test for evaluation of insulin and glucose dynamics in horses. Acta Vet Scand.

[15] de Laat MA, Sillence MN (2017). The repeatability of an oral glucose test in ponies. Equine Vet J.

[16] Ralston SL (2002). Insulin and glucose regulation. Vet Clin North Am Equine Pract..

[17] Öberg J, Bröjer J, Wattle O, Lilliehöök I (2011). Evaluation of an equine-optimized enzyme-linked immunosorbent assay for serum insulin measurement and stability study of equine serum insulin. Comp Clin Pathol.

[18] Warnken T, Huber K, Feige K (2016). Comparison of three different methods for the quantification of equine insulin. BMC Vet Res..

[19] Mercodia AB. Technical Note No: 34-0152: how to convert units when using Mercodia’s animal insulin ELISAs. https://www.mercodia.com/assets/upload/files/TN34-0152%20converting%20insulin%20units%20v%201.pdf. Accessed 05 May 2017.

[20] Fraley C, Raftery AE (2002). Model-based clustering, discriminant analysis, and density estimation. J Am Stat Assoc.

[21] Scrucca L, Raftery AE (2015). Improved initialisation of model-based clustering using Gaussian hierarchical partitions. Adv Data Anal Classif.

[22] Tinworth KD, Wynn PC, Boston RC, Harris PA, Sillence MN, Thevis M, Thomas A, Noble GK (2011). Evaluation of commercially available assays for the measurement of equine insulin. Domest Anim Endocrinol.

[23] Banse HE, McFarlane D (2014). Comparison of three methods for evaluation of equine insulin regulation in horses of varied body condition score. J Equine Vet Sci..

[24] Borer-Weir KE, Bailey SR, Menzies-Gow NJ, Harris PA, Elliott J (2012). Evaluation of a commercially available radioimmunoassay and species-specific ELISAs for measurement of high concentrations of insulin in equine serum. Am J Vet Res.

[25] Frank N, Walsh DM (2017). Repeatability of oral sugar test results, glucagon-like peptide-1 measurements, and serum high-molecular-weight adiponectin concentrations in horses. J Vet Intern Med.

